# Characterization of Atlantic Forest Tucum (*Bactris setosa* Mart.) Leaf Fibers: Aspects of Innovation, Waste Valorization and Sustainability

**DOI:** 10.3390/plants13202916

**Published:** 2024-10-18

**Authors:** Taynara Thaís Flohr, Eduardo Guilherme Cividini Neiva, Marina Proença Dantas, Rúbia Carvalho Gomes Corrêa, Natália Ueda Yamaguchi, Rosane Marina Peralta, Afonso Henrique da Silva Júnior, Joziel Aparecido da Cruz, Catia Rosana Lange de Aguiar, Carlos Rafael Silva de Oliveira

**Affiliations:** 1 Textile Engineering Department, Federal University of Santa Catarina—UFSC, Blumenau Campus, Blumenau 89036-004, SC, Brazil; 2 Chemistry Department, University of Blumenau—FURB, Blumenau 89030-903, SC, Brazil; 3 Graduate Program in Clean Technologies, Cesumar Institute of Science, Technology and Innovation—ICETI, Cesumar University, Maringá 87050-900, PR, Brazil; marina.dantas@unicesumar.edu.br (M.P.D.);; 4 Energy and Sustainability Department, Federal University of Santa Catarina—UFSC, Araranguá Campus, Araranguá 88905-120, SC, Brazil; 5 Center for Biological Sciences, Biochemistry Department, State University of Maringá—UEM, Maringá 87020-900, PR, Brazil; 6 Chemical Engineering Department, Federal University of Santa Catarina—UFSC, Florianópolis 88040-900, SC, Brazil

**Keywords:** natural fibers, tucum fibers, fiber characterization, sustainable materials, industrial crop

## Abstract

This study investigates the fibers of tucum (*Bactris setosa* Mart.), a palm species native to the Atlantic Forest. The fibers manually extracted from tucum leaves were characterized to determine important properties that help with the recognition of the material. The fibers were also subjected to pre-bleaching to evaluate their dyeing potential. The extraction and characterization of these fibers revealed excellent properties, making this material suitable not only for manufacturing high-quality textile products but also for various technical and engineering applications. The characterization techniques included SEM (Scanning Electron Microscopy), FTIR (Fourier Transform Infrared Spectroscopy), TGA (Thermogravimetric Analysis), and tensile strength tests. These analyses showed that tucum fibers possess desirable properties, such as high tensile strength, with values comparable to linen but with a much finer diameter. The fibers also demonstrated good affinity for dyes, comparable to cotton fibers. An SEM analysis revealed a rough surface, with superficial phytoliths contributing to their excellent mechanical strength. FTIR presented a spectrum compatible with cellulose, confirming its main composition and highly hydrophilic nature. The dyeing tests indicated that tucum fibers can be successfully dyed with industrial direct dyes, showing good color yield and uniformity. This study highlights the potential of tucum fibers as a renewable, biodegradable, and sustainable alternative for the transformation industry, promoting waste valorization.

## 1. Introduction

Tucum (*Bactris setosa* Mart.), a palm tree native to the Americas, has been historically used by Indigenous communities for various purposes, including the extraction of fibers to produce fishing nets, decorative ornaments, and binding ropes [1,2,3]. Despite being widely used by native peoples, tucum fibers are currently little known, and their use remains underexplored.

**Figure 1:**
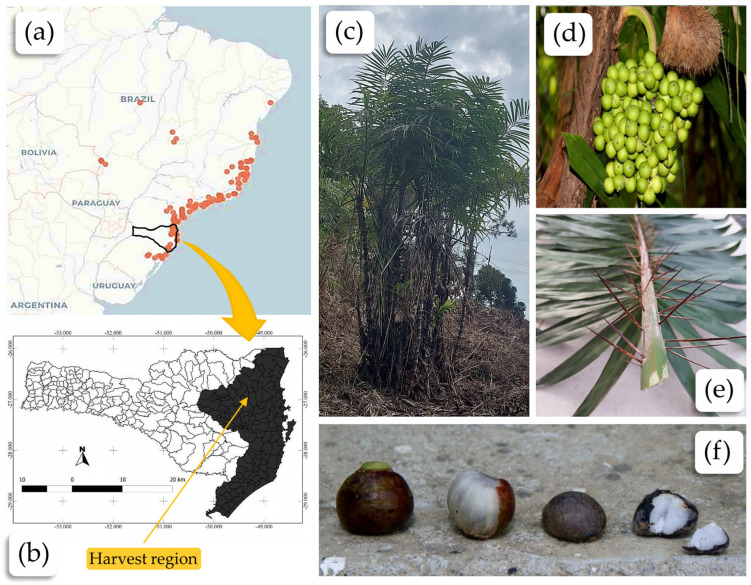
Tucum palm (*Bactris setosa* Mart.): (**a**) records of occurrences of the species *Bactris setosa* Mart. in Brazil (reproduced with permission from [25]); (**b**) map of the geographic distribution of the species *Bactris setosa* Mart. in the Brazilian state of Santa Catarina (Reproduced with permission from [26]); (**c**) clump from which samples were taken; (**d**) green fruit of the tucum palm; (**e**) detail of the thorns on the rachis of the tucum leaf; (**f**) ripe fruit of the tucum palm, from left to right: first, the fruit with its outer skin; next, the same fruit without the skin, exposing the pulp; and finally, the whole and broken almond, from which tucum oil is extracted.

Tucum is a common name for various palm trees of the genus *Astrocaryum* [4] and *Bactris* [5] native to South America, especially Brazil [6,7], Paraguay, and Bolivia (see Figure 1a). The genus *Bactris* is a taxonomic genus that encompasses various species of palms, distributed mainly in tropical and subtropical regions of South America, Central America, and the Caribbean [7]. This genus includes a wide diversity of species adapted to different environments, from humid forests to drier regions such as the Brazilian Cerrado and Pantanal [6,8,9]. The greatest diversity of tucum species is found in the Amazon region [10,11,12,13], while other species are primarily located in the Atlantic Forest [1,14,15]. Some *Bactris* species are notable for their economic, nutritional, and cultural importance [8,16,17,18]. An example is the peach palm (*Bactris gasipaes*), whose fruits are widely consumed and can be used to produce oils [19] and flours [20]. Additionally, part of its stem can be used to produce the heart of the palm [21]. The tucum palm produces plants that provide edible fruits (see Figure 1d,f), are rich in carbohydrates and vitamin A, and are suitable for human and animal consumption [21,22]. The seeds from the tucum fruit (popularly known as tucunzeiro in Brazil) have an almond used for extracting edible oil [23], currently underproduced due to the strong soybean oil market but with potential for biodiesel production [24].

In addition to fruits, the tucum palm has leaves that are discarded as waste, from which some species enable the extraction of leaf fibers [5,10,27] with micrometric thickness, long length, high rupture resistance, and excellent malleability. Valuing this waste can be a true treasure for the manufacturing industry, given the potential uses of the extracted fibers as a vegetable fiber alternative in the production of textile products, such as yarns, fabrics, and nonwovens [25,26,27,28]. Tucum fibers also have innovation potential in the manufacture of composites due to their mechanical properties [10,29], which can provide excellent strength and reinforcement to these materials at a low cost and with zero pollution [30].

The scarcity of literature on the extraction and characterization of tucum fibers of the *Bactris setosa* Mart. type, combined with the limited knowledge of this material’s potential, was a determining factor for carrying out this research. The lack of in-depth studies in this area prevents the efficient exploration of its properties, limiting its use in the textile industry and other sustainable applications. Therefore, this work aims to fill this gap by providing detailed data on extraction methods and the physical, chemical, and mechanical characteristics of tucum fibers. Thus, the goal is to reveal the potential of this natural resource as an alternative for producing innovative and sustainable textile products.

## 2. Results

The manual extraction of the tucum fiber proved to be effective, demonstrating the feasibility of the technique, considering that it allowed up to five extractions from the same leaflet. Manual extraction is quite laborious, presenting an opportunity for innovation through the development of an efficient mechanical extraction system. The manually extracted fibers have a crimped appearance, which may be related to the more intense mechanical action of the manual method. The advantage of the manual extraction technique is that it does not use chemicals and, therefore, can be considered more sustainable when compared to other natural fibers or man-made fibers.

### 2.1. SEM and EDS Analyses of Raw Fiber

**Figure 2:**
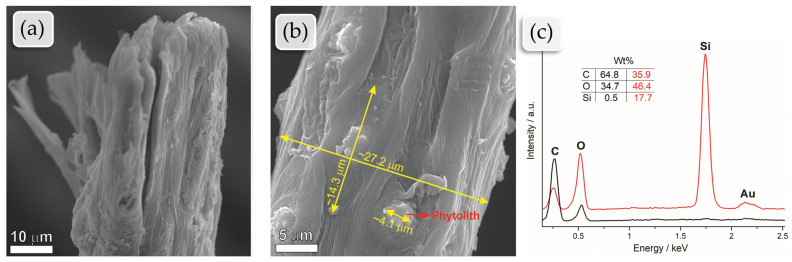
SEM analysis of raw tucum fibers: (**a**) panoramic view of the cross-section of a tucum fiber from its end, magnification of 3600×; (**b**) longitudinal view of tucum fibers, magnification of 7000×; (**c**) EDS analysis of tucum fiber, the black curve represents the EDS spectrum of the surface in a region without phytoliths, while the red curve represents the spectrum from the region containing the phytolith.

**Figure 3:**
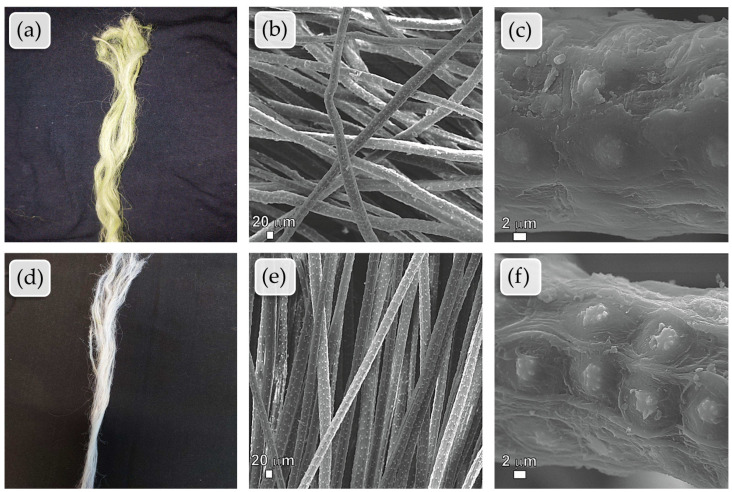
SEM analysis of manually extracted raw tucum fibers and pre-bleached fiber: (**a**) photographic image of a bundle of raw fibers (before pre-bleaching); (**b**,**c**) longitudinal view of the manually extracted raw fiber, with magnifications of 300× and 5800×, respectively. (**d**) photographic image of a bundle of pre-bleached fibers; (**e**,**f**) longitudinal view of the pre-bleached fiber with H_2_O_2_, with magnifications of 295× and 5300×, respectively.

The SEM analyses of tucum fibers reveal details of the fiber morphology, as shown in Figure 2 and Figure 3. Figure 2b shows many bright spots, which can be observed along the entire length of the fibers. These spots were also observed in the analyses of other researchers who studied tucum fibers from different species, such as [4,31,32]. The observed spots are phytoliths, which are microscopic bodies made of silica (silicon dioxide) that accumulate in the epidermal cells of the plant (it can also be seen in Figure 3c), which is the outermost layer of the leaf or stem [4,33].

Phytoliths play a crucial role in enhancing the hardness and strength of plant fibers, making them less appealing to pests and more resistant to gnawing, thereby serving as a defense mechanism against predation. They also contribute to the rigidity and strength of the plant, which is particularly important for plants that need to withstand harsh weather conditions [32,34]. Furthermore, phytoliths help maintain the integrity of the fiber, preventing it from breaking or collapsing easily under pressure or aggressive external conditions. They also aid in water regulation in the plant, facilitating water retention in plant cells and efficient water distribution through the fibers [35]. Throughout the plant’s life, phytoliths act as a barrier against pathogens, hindering the penetration and entry of fungi, bacteria, and other infectious agents, thereby increasing the durability and longevity of the leaves [29,34].

The EDS analyses in Figure 2c confirm the high silicon concentration in the bright spots and its absence in the other regions, where only carbon and oxygen are present. The gold peaks in the EDS spectrum are due to the sample preparation for the analysis, as they were coated with the noble metal to be inert and conductive.

Figure 2b shows the measurements obtained by the free-use software Image-J Version 1.54. By analyzing various micrographs and measurement points, it was determined that the average diameter of tucum fibers is 28.3 µm. Additionally, the average length of the plant cells around the phytoliths was 15.1 µm, while the average diameter was 3.9 µm. According to [36], human hair diameter varies from 10 µm to 120 µm. Tucum fibers have, thus, a diameter similar to human hair, reaching up to 1/3 of that diameter. The fineness of the fiber is undoubtedly a significant advantage in terms of the textile processability of the material, its final application, and product quality. The fineness of the fiber can also be quite interesting for other applications in materials engineering, such as in the production of composites.

Figure 3 shows that the fibers have a slender shape, and although they appear smooth to the naked eye, the micrographs reveal that the surface is quite rough with very prominent grooves. The images compare manually extracted raw tucum fibers (Figure 3a) and tucum fibers pre-bleached with H_2_O_2_ (Figure 3d); the images show that there was a significant lightening of the fiber due to the pre-bleaching process, which in turn removed the fiber’s natural pigmentation, making it white. The other images in the figure do not reveal significant changes in the fiber between the different conditions, indicating no deterioration or improvement in the material’s appearance due to the pre-bleaching. It is important to note that the regions of the phytoliths vary slightly in each condition. In Figure 3c, the phytoliths do not appear completely superficial as in the pre-bleached fiber (see Figure 3f); in the raw fiber, they give the impression of being covered by layers of lignocellulosic material that partially envelop them. This is consistent considering that the fiber was treated with H_2_O_2_ and underwent an oxidizing treatment for bleaching, as the oxidizing action of the peroxide may have removed the outermost layers of deposited lignins, pectins, and hemicelluloses covering the phytoliths, leaving them more exposed.

### 2.2. Length and Regain Analyses

**Figure 4:**
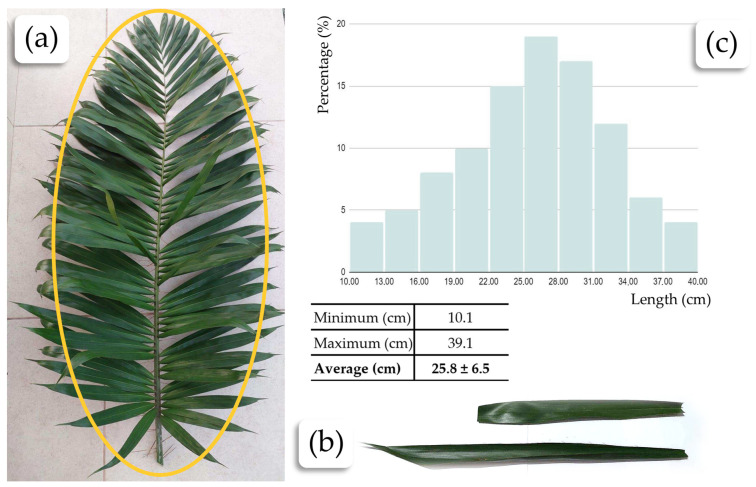
Determination of the average fiber length: (**a**) shape of the tucum palm leaf × length of the leaflets; (**b**) variation in leaflet sizes depending on the region of the leaf; (**c**) histogram of fiber length distribution of the analyzed sample.

One hundred tucum-extracted fibers were measured using a millimeter ruler. The distribution of the fiber lengths was organized in a histogram in Figure 4c. The average fiber length was 25.8 cm. The fibers have a wide variation in size, which can be explained by the shape of the tucum palm leaf itself, which is elliptical, as shown in Figure 4a. The leaflets at the ends of the leaf are smaller compared to those in the central region.

For determining the regain, five samples of tucum fibers were analyzed. Table 1 presents the average value, standard deviation, and coefficient of variation of the measurements.

The measured regain is consistent with the studies by [4,31] on the tucum fiber extracted from the species *Astrocaryum chambira* Burret, which reported a regain of 10.0%. Similarly, the regain found in the present study is comparable to the one of other cellulosic fibers, as shown in Table 2.

A garment made of low hygroscopic fibers cannot absorb the perspiration of its wearer, presents low thermal insulation, and tends to accumulate static charge, causing physiological and esthetic discomfort [37,38,39]. Therefore, it is possible to infer that the regain value found for tucum fibers, being similar to that of cotton, means that tucum fibers are similarly hygroscopic and, thus, can provide similar physiological comfort if used in clothing. This characteristic can be advantageous in textile applications, as fibers with high regain tend to provide adequate physiological comfort by absorbing sweat and maintaining a feeling of freshness for the wearer. Additionally, a high regain value is synonymous with high hydrophilicity, indicating a probable dye affinity with ionic dyes and finishes [40].

### 2.3. FTIR Analysis of Raw Fiber

**Figure 5:**
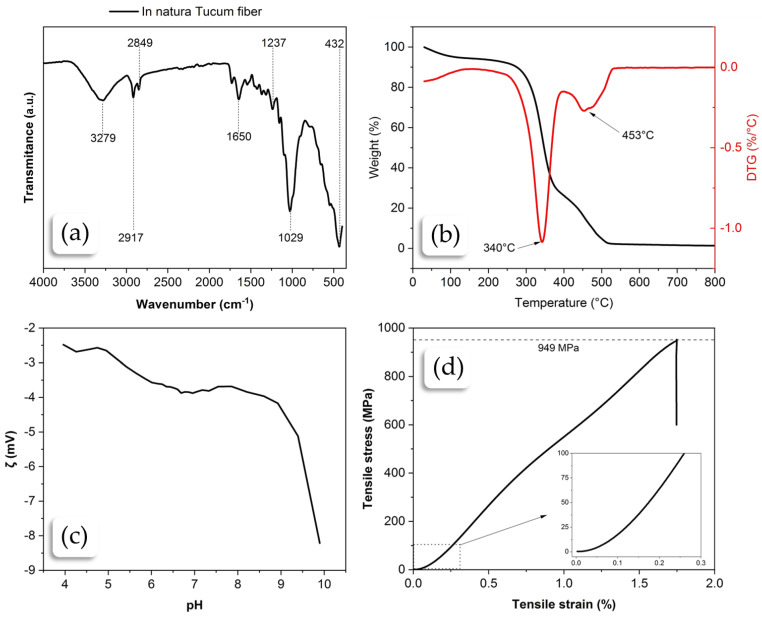
Characterization analysis of manually-extracted tucum fibers: (**a**) FTIR analysis of the raw tucum fiber; (**b**) TGA-DTG analysis of the raw tucum fiber; (**c**) Zeta Potential analysis of the raw tucum fiber; (**d**) stress–strain analysis of the raw tucum fiber.

The spectrum presented in Figure 5a shows typical characteristics of plant materials, such as cellulose, hemicellulose, and lignin. Therefore, the leading bands observed confirmed the analysis of the tucum fiber. The peaks observed between 3600 cm^−1^ and 3200 cm^−1^ can be attributed to the stretching vibrations of hydroxyl (O-H) bonds [41]. It is common in compounds containing water or hydroxyl groups, such as cellulose, hemicellulose, and lignin, in plant fibers. The presence of these bands confirms the hydrophilic nature of the plant fibers. The most prominent peaks in the region between 3000 cm^−1^ and 2800 cm^−1^ correspond to the stretching vibrations of C-H bonds, indicating the presence of carbon chains formed by carbon and hydrogen bonds, which is common in carbohydrate polymers such as cellulose and hemicellulose. The band at 1650 cm^−1^ may be associated with water deformation [42,43,44]. The region between 1750 cm^−1^ and 1500 cm^−1^ showed bands that may be associated with the stretching of carbonyl (C=O) bonds, which may be present in acetyl groups of hemicellulose or esters. They can also indicate aromatic groups, such as those found in lignins, characterized by C=C stretching vibrations. The region between 1500 cm^−1^ and 1200 cm^−1^ also presents bands of C-H bending vibrations and C-O stretching, common in ethers and alcohols, and are characteristic of cellulose and hemicellulose structures. The bands observed between 1200 cm^−1^ and 900 cm^−1^ in plant materials are often attributed to various stretching and bending vibrations of bonds found in polysaccharides such as cellulose, starches, and sugars, indicating the presence of β-(1→4) glycosidic bonds [42].

### 2.4. TGA-DTG Analysis of Raw Fiber

Raw fibers’ thermogravimetric analysis (TGA) was conducted between 30 °C and 800 °C in a synthetic air atmosphere. Based on the results obtained, it was possible to identify several stages of thermal degradation, as shown in Figure 5b. The first stage of mass loss occurs approximately between 30 °C and 150 °C. In the literature, this phase is frequently observed in lignocellulosic materials. This stage is generally attributed to the loss of light volatile organic compounds and mainly to the dehydration of the fiber [45]. In this phase, an approximate mass loss of 5.4% was observed. The main reason for the mass decrease can largely be attributed to the loss of moisture from the fiber, that is, the free water adsorbed on the fiber surface and its structural water. The second degradation stage occurred between 150 °C and 400 °C [43]. In this phase, a significant mass loss of about 66.2% was observed, attributed to the degradation of hemicellulose and cellulose. Hemicellulose degrades between 200 °C and 300 °C, while cellulose degrades between 300 °C and 400 °C. Hemicellulose, having an amorphous structure and being less thermally stable, degrades first. The maximum thermal degradation peak for the raw fibers was at 347 °C. Several studies which investigated the thermal degradation of plant fibers in this temperature range, associated the mass loss with the breakdown of glycosidic bonds and the thermal depolymerization of cellulose. This stage produces most pyrolysis products, such as l-glucose, tar, ketones, alcohols, esters, aldehydes, and CO [43,45,46]. The third degradation phase was observed between 400 °C and 800 °C, consistent with the degradation of lignin and the formation of more thermally stable carbonaceous residues. The mass loss in this phase was about 22.7%. Lignin has a complex structure and is more heat-resistant than cellulose, degrading over a broader temperature range (250–800 °C). The literature indicates that this phase forms aromatic compounds and charcoal, which are more resistant to thermal degradation. At the end of the process, a residual degradation mass of approximately 3.9% was observed at 800 °C; since the atmosphere is oxidizing, this residue may have possibly formed mainly from SiO_2_. TGA studies on plant fibers, such as coconut fiber, bamboo, flax, and other lignocellulosic fibers, show similar thermal degradation profiles [47]. These studies demonstrate a direct correlation between the chemical structure of the plant fiber and the degradation peaks observed in the TGA. Fibers with higher lignin content show greater thermal stability.

### 2.5. Zeta Potential Analysis of Raw Fiber

The graph in Figure 5c shows the variation of the Zeta potential (in millivolts) as a function of the pH of the tucum fiber. In the acidic range (pH 0–6), the Zeta potential of the fiber is relatively stable, varying between −2 mV and −4 mV. Starting from pH 6, the Zeta potential of the fiber begins to decrease gradually, reaching about −4 mV to −5 mV around pH 7. This trend continues until pH 9, where a more pronounced drop in Zeta potential occurs. After pH 9, the Zeta potential drops drastically, reaching values of approximately −9 mV at pH 10. The analysis does not identify the isoelectric point where the net surface charge is zero.

In summary, the presence of a negative Zeta potential in acidic pH indicates the presence of ionizable functional groups, such as carboxyl or hydroxyl groups, in the cellulosic fiber. These groups remain ionized even in very acidic conditions [48,49]. In alkaline pH, the cellulosic fiber acquires a greater negative charge due to the deprotonation of the functional groups. This behavior is typical of materials containing carboxyl and hydroxyl groups that ionize at high pH [48,49]. Understanding the behavior of Zeta potential as a function of pH is crucial for optimizing chemical treatment processes, such as surface modification of the fiber to improve its adsorption properties or compatibility with polymer matrices. In formulations where the fiber is used, adjusting the pH to regions where the surface charge is significantly negative can improve dispersion and process stability, thereby enhancing the practical applications of the research.

### 2.6. Mechanical Tensile Analysis of Raw Fiber

The graph in Figure 5d illustrates a tucum plant fiber’s stress versus strain behavior under mechanical strength testing. This graph is essential for understanding the fiber’s mechanical properties, which are fundamental for applications in composites and other industrial uses. The strain axis represents the fiber’s relative deformation, measured as a percentage. Initially measured in kgf/mm^2^, the stress axis was converted to MPa and represented the stress applied to the fiber.

The analysis of the stress–strain curve between 0 and 0.25% strain shows that the stress increases almost linearly with the strain, indicating an initial elastic behavior. This suggests that the tucum fiber deforms elastically under small stresses, returning to its original shape when the stress is removed. Considering the entire stress range up to rupture, the trend line drawn from the numerical analysis data showed that the R^2^ is approximately 0.96, meaning that a linear function well represents this range. This indicates that the mechanical behavior of the fiber exclusively follows Hooke’s law. That is, the analyzed fiber does not exhibit plasticity; it only shows elastic behavior. Therefore, upon reaching the limit of its elasticity, the fiber breaks and does not continue to deform, unlike synthetic, highly ductile fibers. The modulus of elasticity was calculated from the slope of the line, yielding a value of 0.56 GPa.

In the region close to a 1.75% strain, the stress reaches a peak of approximately 949 MPa. This point represents the maximum strength of the fiber before the onset of failure. The tensile strength value presented is an average of 10 measurements, which showed a deviation of approximately ±14% from the mean value. The ability to withstand such a level of stress indicates that the tucum fiber has high tensile strength, surpassing fibers like cotton (~400 MPa) [50], sisal (~650 MPa) [51], and silk (~750 MPa) [52], reaching strength values close to that of flax (~1200 MPa) [53], while being much finer and more delicate than flax. In the region of 1.75% strain, there is an abrupt drop, indicating the sudden failure of the fiber. This behavior suggests a brittle mechanical behavior, where the fiber breaks almost instantly after reaching its maximum strength. After failure, the stress stabilizes at low values, close to zero. This indicates that the fiber no longer supports significant loads after the rupture.

### 2.7. Dyeing Test of the Pre-Bleached Fiber

The tucum fibers prepared through the pre-bleaching process showed a distinct whitening, demonstrating a considerable color change, with a clean and clear base, making it suitable for the dyeing stage. Thus, it can be stated that the auxiliary products used in the pre-bleaching process were effective in aiding the cleaning and whitening of the substrate. The raw and pre-bleached substrates were evaluated by reflectance spectroscopy, and their colorimetric coordinates can be seen in Table 3.

Analyzing the results, an increase in the L* coordinate is observed for the fibers subjected to pre-bleaching, indicating a higher brightness of the color. Regarding the a* coordinate, an increase is observed, meaning that the substrate acquired a less greenish tone. As for the b* coordinate, its decrease indicates a reduction in the yellowish color of the sample. The whiteness degree of the sample was then verified according to the Berger scale. The value found is 30.98, indicating that the obtained white is yellowish, confirmed in the visual analysis of Figure 3d.

**Figure 6:**
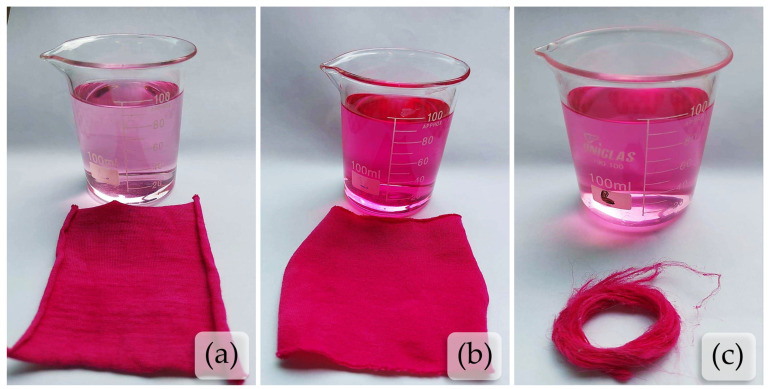
Comparison of dyed samples and their respective residual dye baths: (**a**) cotton fabric; (**b**) viscose fabric; (**c**) tucum fiber.

After pre-bleaching, the fibers were dyed. The dyeing was performed on samples of tucum fibers and samples of CO and CV, using direct dye Pink Tricel NG-LRB at concentrations of 1% o.w.m. The dyed samples can be seen in Figure 6, and the obtained colorimetric coordinates are presented in Table 4.

It is possible to verify that the viscose knit sample (CV) showed a lower color intensity compared to the tucum and cotton (CO) samples. This can be explained by the fact that the sample is not composed of 100% CV, also containing elastane, which is not chemically capable of being dyed by the direct dye. Therefore, only the data from the cotton and tucum samples will be considered for comparison.

It is evident that both the cotton fabric and the tucum fibers obtained a similar visual appearance. However, the spectral data show that the L* coordinate decreased from the standard CO sample to the tucum, indicating lower color brightness, i.e., the tucum is darker. Regarding the a* coordinate, an increase is observed, meaning that the tucum acquired a more reddish tone. As for the b* coordinate, its increase indicates a reduction in the bluish color of the sample. Thus, from the K/S value, it is possible to identify that the tucum fiber has a higher color strength when compared to the cotton sample. However, due to the substrates being in different forms (fabric and fiber), the results are inconclusive. This underscores the importance of your role in the research process, as further tests using the same substrate form, i.e., fiber–fiber or fabric–fabric, are necessary for conclusive results.

Overall, it can be concluded that the tucum fiber, a cellulosic fiber, can likewise be dyed using direct dyes (see Figure 6c), following a dyeing mechanism similar to the cotton’s mechanism. Notably, no stains or lack of color equalization were observed in all dyed samples.

## 3. Discussion

The present study revealed that fibers from tucum, derived from *Bactris setosa* Mart., could be a great material for mechanical reinforcement applications, as evidenced by the excellent tensile strength demonstrated in the tests. The fibers are also extremely fine, which can be advantageous for precise applications that require simultaneously high degree of detail, lightness, and strength. The limited literature on the use of this fiber for technical applications has shown promising results for its use in composites.

The use of the tucum fiber in engineering composites has gained prominence, especially in Brazil, where its potential to enhance the properties of these materials is being explored [54]. Studies have shown that the tucum fiber can improve the characteristics of composites, enabling new industrial applications. An example is the work of Monteiro et al. [55], who compared tucum and mallow fibers in polyurethane composites. They found that the tucum fiber has higher crystallinity and thermal stability compared to mallow fiber and superior performance in flexural tests, albeit with higher water absorption, suggesting good interaction between the fibers and the matrix. Another study by Monteiro et al. [56], analyzed the mechanical and ballistic properties of composites made from epoxy reinforced with tucum fiber. Although these composites showed inferior performance compared to some materials, such as ramie and banana fibers, their performance was reasonable compared to other natural fibers, and the use of tucum fiber brought advantages in terms of sustainability and lower cost. Additionally, research by Panzera et. al. [57], explored using tucum powder in composites with recycled polypropylene. Despite decreased mechanical performance in tensile, flexural, and impact tests, the composites showed higher compression resistance and better flame retardancy, mainly when higher concentrations of tucum were used. These studies indicate that, although the mechanical properties of the tucum fiber are not as robust as those of other natural fibers like sisal or curauá, it has significant potential for use in engineering applications, especially with the application of surface treatments to improve interfacial adhesion and, consequently, the properties of the final composites. With the growing interest in tucum fiber, more studies will likely emerge, further exploring its potential.

Another aspect of the conscious exploration of tucum fiber concerns sustainability [58] and waste valorization. The close dependence of indigenous peoples on the environment represents a unique form of society-nature relationship. This relationship is recognized for its low environmental impact, thus being capable of promoting biodiversity conservation [30]. In this context, in the face of the environmental crisis that the modern world is experiencing, the way of life and knowledge of these indigenous populations have been considered important global environmental preservation tools. Within this scenario, the environmental impacts of the textile and fashion industry are present in all market segments, ranging from marketing, sales, and transportation to the final phase of textile product use by the consumer and its disposal [30]. Recent research has highlighted how mass cotton production can harm soil health, air quality, and the high-water consumption required for its cultivation and processing [30,59]. Additionally, there is growing concern about the environmental and health problems caused by micro(nano)plastics released during the natural degradation of synthetic textiles, such as polyester, nylon, and other plastic materials [60,61]. The most critical environmental issues in the fashion industry include high energy and water consumption [62], the use of toxic chemicals [63], and the generation of large volumes of waste. Moreover, various social issues are also common in the contemporary fashion industry, such as poor working conditions, child labor, and unfair wages in developing countries. Alternative plant-based fibers, whether natural or synthetic, have become increasingly attractive options in the textile industry due to their abundance, low environmental impact, and suitable technological properties for specific applications and innovation. This movement drives increased research on using these fibers and their waste, motivated by social, economic, and sustainability considerations.

## 4. Materials and Methods

The proposed methodology is divided into three stages. The first stage consists of collecting tucunzeiro leaves, selecting the pinnate leaflets and then manually extracting the fibers. The second stage was the characterization of the raw fibers to determine their composition and some of their characteristics and properties. Finally, the third stage was the dyeing test of the pre-bleached fibers for comparison with the dyeing of cellulose fibers of natural origin, in this case, cotton, and of artificial origin, in this case, viscose.

### 4.1. Materials

A Emulgator agent, under the name Bioten MK T-SFE, was purchased from MK Química, Brazil. Hydrogen peroxide 50% b.w., glacial acetic acid PA ACS, and sodium chloride PA were obtained from CRQ Química, Brazil. Sodium hydroxide (pearly solid) was provided by Sciavicco Ltd., Brazil. Industrial textile direct dye under the commercial name Pink Tricel NG-LRB (C.I. No. Direct Red 31) was supplied by TMX Comércio, Importação and Exportação Ltd., Brazil. Color Química do Brasil Company, kindly supplied the wetting detergent Colorswet DTU-M, used to dye the fibers. 100% knitted fabric and 98% rayon viscose and 2% elastane pre-bleached knitted fabric were donated by industries in the of Blumenau-SC, Brazil. The fabrics were used to compare with the color yield of the dyeing of pre-bleached tucum fibers.

#### Plant Material

For this study, fibers were obtained from new and mature leaves (open or pinnate leaves) of the tucum palm (*Bactris setosa* Mart.), collected in the Vila Itoupava neighborhood, in the city of Blumenau-SC, Brazil, with coordinates 26°42′22.3″ S, 49°07′35.0″ W.

### 4.2. Experimental Procedure

#### 4.2.1. Extraction of Tucum Fibers

**Figure 7:**
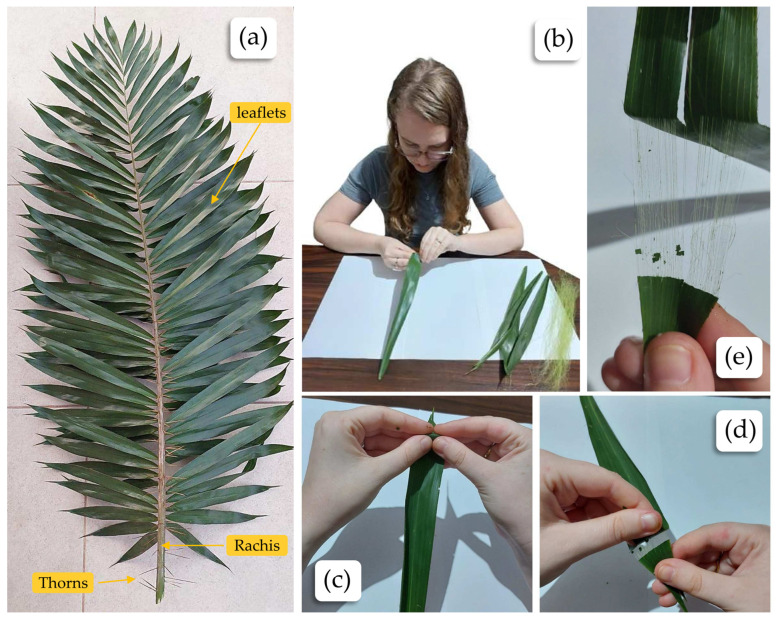
Steps of the manual process of fiber extraction from the leaves of the genus *Bactris setosa* Mart.: (**a**) tucum leaf; (**b**) manual process of tucum fiber extraction; (**c**) the leaflets were removed from the rachis, then a fold was made near the tip of the leaflet, creating a crease; (**d**,**e**) after folding the crease, the epidermis of the leaflet was pulled, promoting the release of the fibers along the entire leaflet, and this process could be repeated up to five times for complete extraction.

The tucum fibers were manually extracted (from leaflets) following the ancient method used by local inhabitants who maintained the practice learned from their ancestors. Figure 7 shows the fiber extraction process. Figure 7a shows the tucunzeiro leaf. This type of leaf is called a pinnate leaf or pinnatifid leaf, is characterized by having leaflets arranged on either side of a central axis, known as the rachis.

#### 4.2.2. Pre-Bleaching of Raw Tucum Fibers

**Figure 8:**
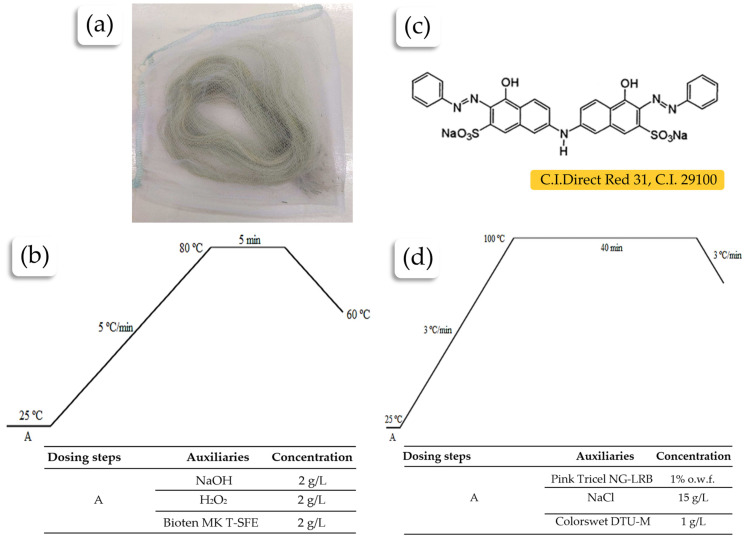
Treatment parameters of the raw fibers: (**a**) placement of the fibers in a small tulle fabric pouch; (**b**) temperature curve of the pre-bleaching process; (**c**) molecule of the dye used in the dyeing of fiber and fabric samples; (**d**) temperature curve of the dyeing process.

After extraction, the fibers underwent an oxidative pre-bleaching process using hydrogen peroxide 50% b.w. and sodium hydroxide 50%, which was used to adjust the pH (~11) and for oxidative activation. The sodium hydroxide also promoted the saponification of natural greasy impurities from the fibers and helped remove other impurities and natural pigmentation. The main function of pre-bleaching is to prepare the fibers for the subsequent dyeing stage. For a more efficient cleaning, the Bioten MK T-SFE emulsifier (MK Química, Boa Vista, RS, Brazil), with its detergent action and low foam formation, was added. The process conditions followed methods described by [64], with the chemical auxiliaries dissolved in deionized water. Before the process, a bundle of fibers was made and placed in a “small tulle fabric pouch” (see Figure 8a) to prevent the entanglement of loose fibers in the bath during the process, which would make further handling more difficult. The pre-bleaching process was carried out using the HT IR Dyer Texcontrol 2200 (Texcontrol, São Paulo, SP, Brazil) at a temperature of 80 °C for 5 min. The bath ratio used was 1:100, meaning that for every 1 g of fiber, 100 mL of solution was used (See Figure 8b). After pre-bleaching, the residual bath was discharged at 60 °C, and the samples were rinsed in a 2% acetic acid solution (*v*/*v*) to neutralize the pH. After neutralization, a rinse with deionized water was performed, and the sample was taken to an oven for drying at 80 °C. Then, to assess the change in color and the degree of whiteness, the samples were analyzed using a Datacolor^®^ Spectrum 500 (Datacolor, Barueri, SP, Brazil) reflectance spectrophotometer.

#### 4.2.3. Dyeing of Pre-Bleached Tucum Fibers

To verify the dyeing behavior of tucum fibers, the pre-bleached fibers were dyed using the direct dye Pink Tricel NG-LRB at a proportion of 1% weight of the material (o.w.m.). The tests were carried out in a HT IR Dyer TC 2200 dyeing machine, with each sample weighing 0.5 g and a bath ratio of 1:20. The dispersant used was Colorswet DTU-M, and the dyeing pH was maintained at 7.0. As in the pre-bleaching process, the fibers were placed in a small tulle fabric pouch for dyeing. For comparative purposes, dyeing was also carried out on a sample of 100% Cotton (CO) fabric and a sample of viscose (CV) fabric with elastane. The dyeing parameters are presented in Figure 8d.

### 4.3. Characterization

The morphology of the raw and pre-bleached fibers was characterized by scanning electron microscopy (SEM). SEM images were collected using Tescan equipment, utilizing a secondary electron detector and operating at 20 kV, with an Oxford energy dispersive spectroscopy (EDS) system attached. The magnifications used for these analyses were up to 7000×. The fibers were gently attached to a conductive carbon tape placed on the holder and then metalized with Au to provide a conductive surface.

The length of the tucum fibers was determined manually using a millimeter ruler, following the method used by [31].

Fiber regain refers to the amount of moisture a fiber can absorb from the environment relative to its dry weight. This parameter is expressed as a percentage and is essential for characterizing the hygroscopic properties of textile fibers. The fiber regain is calculated by Equation (1). The method used to obtain the regain was adapted from the methods used by [31,40]. The moisture content was determined by weighing the fiber samples, which were conditioned for 24 h at 20 °C and 65% relative humidity. The experiment was conducted via drying in an oven at 70 °C until a constant weight was reached. The experiment was performed in quintuplicate using raw, pre-bleached, and dyed fibers. After obtaining the values, they were applied to Equation (1) to find the regain percentage.

(1) $$Regain\;\left(\%\right)=\left(\frac{{Weight}_{Wet}-{Weight}_{Dry}}{{Weight}_{Dry}}\right)\times 100$$

To analyze the fiber composition and its surface functional groups, Fourier-Transform Infrared Spectroscopy (FTIR) was used, covering wavelengths from 4000 to 400 cm^−1^, with an INVENIO^®^ (Bruker) device, recording 20 scans in attenuated total reflection (ATR) mode with a resolution of 4 cm^−1^.

The thermal behavior of the samples was investigated by thermogravimetry (TGA) using a Jupiter STA-449 F3 (Netzsch) device. The samples were heated from room temperature to 800 °C at a heating rate of 10 °C/min under a synthetic air atmosphere with a 20 mL/min flow rate. A synthetic air cylinder supplied by AirGas with a composition of 20% ± 0.5% oxygen and 79% ± 0.5% nitrogen, which closely mimics the natural atmospheric composition was used. The purity of the gas was 99.999%, ensuring a controlled and consistent atmosphere during the heating process.

The surface charge of the raw fiber was investigated by Zeta Potential measurements using an Anton-Paar SurPASS 2 device. The analyses were carried out by varying the pH from 4 to 10. The mechanical tensile strength tests were performed with a TA HD plus Texture Analyzer (Stable Micro Systems) device using a 50 kg load cell. The tensile testing was performed on individual fibers. Each fiber was tested separately to ensure that the reported mechanical properties accurately reflect the behavior of a single fiber. The test was carried out on 10 individual fibers from different leaves of similar lengths.

## 5. Conclusions

The main objective of this study was to investigate tucum fibers obtained from the species *Bactris setosa* Mart. to evaluate their feasibility and potential application in the textile industry. The research was motivated by growing concerns about the negative environmental impacts associated with the intensive use of synthetic fibers in the textile industry and the need to find more sustainable alternatives. Tucum fiber stands out for its promising characteristics of length, fineness, and mechanical properties, as well as being a renewable and biodegradable raw material, which can significantly contribute to reducing environmental impact.

The results of tucum fiber extraction highlighted the effectiveness of the manual method, which, although more labor-intensive, proved to be more sustainable by not using chemicals [63]. The pre-bleaching process was effective in cleaning and whitening the fibers, adequately preparing them for dyeing, which was successfully carried out using direct dye, showing tucum fiber’s dye affinity with direct dye, similar to cotton.

A mechanical properties analysis revealed that tucum fibers have high tensile strength, comparable to some high-performance synthetic and natural fibers. The fiber also exhibited desirable elastic behavior, returning to its original shape after the load was removed. However, its brittle nature upon failure should be considered in application design.

The viability of tucum fibers for textile use was demonstrated not only by their physical and chemical properties but also by their ability to be dyed and their mechanical strength. The research concludes that tucum fiber is a promising alternative to other natural fibers such as cotton and flax, being suitable for spinning and weaving activities, and contributing to more sustainable practices in the textile industry. Due to the limited studies on the species, precise information on the plant’s life cycle, growth characteristics, and seasonality of leaf production was not obtained, which are essential aspects for understanding harvest availability, necessitating more in-depth studies in this regard.

Finally, this study paves the way for future research that can optimize the extraction and processing methods of tucum fibers and explore their applications in new textile products, composites, and other engineering materials. It is believed that adopting natural fibers such as tucum in the textile industry can reduce dependence on non-renewable resources and promote greater sustainability and innovation in the sector.

## Figures and Tables

**Table 1:** Determination of the regain of raw tucum fiber.

	Initial Mass (g)	Dry Mass (g)	Regain
Mean	0.4614	0.4215	9.45%
Standard deviation	0.06	0.05	0.67%
Coefficient of variation	12.31%	12.09%	7.13%

**Table 2:** Regain of commercial cellulosic fibers.

Fiber	Regain (%)
Non-mercerized cotton	8.5%
Mercerized cotton	10.5%
Linen	12.0%
Jute	13.8%
Hemp	12.0%
Rami	12.0%
Sisal	10–12.0%

**Table 3:** Colorimetric coordinates for raw and pre-bleached tucum fiber.

Sample	L*	a*	b*	Color
Raw fiber	64.3	−6.3	25.1	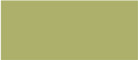
Pre-bleached	85.2	0.4	8.6	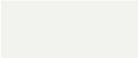

**Table 4:** Colorimetric coordinates of the different dyed substrates.

Sample	L*	a*	b*	K/S	Color
Cotton	45.0	53.1	−7.9	128.2	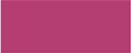
Viscose	50.4	53.6	−8.9	86.1	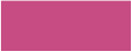
Tucum	43.4	54.4	−2.8	161.2	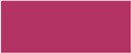

## Data Availability

The data presented in this study are available in this paper.
